# Quantification of oxygen consumption in head and neck cancer using fluorescent sensor foil technology

**DOI:** 10.3389/fonc.2024.1002798

**Published:** 2024-02-08

**Authors:** Magdalena Stocker, Alexandra Blancke Soares, Gregor Liebsch, Robert J. Meier, Martin Canis, Olivier Gires, Frank Haubner

**Affiliations:** ^1^ Department of Otorhinolaryngology, Head and Neck Surgery, University Hospital, Ludwig Maximilians University (LMU) Munich, Munich, Germany; ^2^ PreSens Precision Sensing GmbH, Imaging Solutions, Regensburg, Germany

**Keywords:** tumor hypoxia, HNSCC, tumor imaging, head and neck cancer, oxygen consumption

## Abstract

**Introduction:**

Head and neck squamous cell carcinoma (HNSCC) patients suffer from frequent local recurrences that negatively impact on prognosis. Hence, distinguishing tumor and normal tissue is of clinical importance as it may improve the detection of residual tumor tissue in surgical resection margins and during imaging-based surgery planning. Differences in O_2_ consumption (OC) can be used to this aim, as they provide options for improved surgical, image-guided approaches.

**Methods:**

In the present study, the potential of a fluorescent sensor foil-based technology to quantify OC in HNSCC was evaluated in an *in vitro* 3D model and in situ in patients.

**Results:**

*In vitro* measurements of OC using hypopharyngeal and esophageal cell lines allowed a specific detection of tumor cell spheroids embedded together with cancer-associated fibroblasts in type I collagen extracellular matrix down to a diameter of 440 µm. Pre-surgery in situ measurements were conducted with a handheld recording device and sensor foils with an oxygen permeable membrane and immobilized O_2_-reactive fluorescent dyes. Lateral tongue carcinoma and carcinoma of the floor of the mouth were chosen for analysis owing to their facilitated accessibility. OC was evaluated over a time span of 60 seconds and was significantly higher in tumor tissue compared to healthy mucosa in the vicinity of the tumor.

**Discussion:**

Hence, OC quantification using fluorescent sensor foil-based technology is a relevant parameter for the differentiation of tumor tissue of the head and neck region and may support surgery planning.

## 1 Introduction

Standard treatment of head and neck tumors is based on surgical therapy with adjuvant radio (chemo) therapy, if necessary (1–3). The prerequisite for surgical treatment is the resectability of the tumor in the absence of distant metastases, as cases staged M1 are preferably subjected to systemic treatment. In the context of surgical resection, complete removal of tumor tissue, typically with a minimum resection margin of 5 mm, is of considerable prognostic importance (4–6). Complete removal of the tumor is currently based on intraoperative evaluation by the surgeon and histopathological assessment of frozen sections taken intraoperatively. The assessment of the extent of resection is thus dependent on the experience of the respective surgeons and pathologists. Despite an assessment as R0 in the final histological report of intraoperative frozen sections (*in sano*, no residual tumor cells detectable), minimal residual disease (MRD) must be assumed based on the frequency of local relapses (7). Even in the case of an R0 resection, 10-30% of patients will be diagnosed with a local recurrence within less than three years after treatment. Therefore, additional tools for the assessment of resection margins are in great demand, which could help identifying residual tumor cell depositions in R0-resections and potentially prevent local recurrences (8). Additionally, taking tissue samples from the correct regions for frozen section diagnosis is also dependent on the expertise of the surgeon. An objectifiable imaging method that indicates possible residual tumor tissue *in situ* intraoperatively could result in a considerable facilitation in the decision of the extent of resection and thus in optimized therapy for the patient with a presumably better prognosis. This refers to both, a sufficient tumor resection and to an avoidance of unnecessarily large resection margins with the ensuing increase in morbidity.

Several aspects of tumor biology have been assessed as sources of markers to improve resection margin recognition. These include the antibody-mediated detection of tumor-associated antigens such as the epidermal growth factor receptor EGFR and the epithelial cell adhesion molecule EpCAM in combination with near-infrared dyes (9, 10). Confocal laser endomicroscopy, second and third harmonic generation, Raman spectroscopy, and other technologies are under evaluation to address differences in morphology and collagen distribution between normal and malignant tissue (11). Endomicroscopic methods can be combined with 5-aminolevulinic acid (5-ALA)-induced protoporphyrin IX (PPIX) fluorescence, which accumulates more strongly in tumors (12). A meta-analysis of the sensitivity and specificity of various methods of non-invasive imaging methods for oral cancers has been summarized recently by Mendonca et al. (13).

Alternatively, metabolic changes in malignant cells may be harnessed to differentiate normal and tumor areas. From the 1920’s on, the Warburg effect has been widely discussed as a major driving force in tumor initiation and progression. Tumor cells use aerobic glycolysis for ATP generation, despite the availability of oxygen and thus of the more efficient energy supply through mitochondrial oxidative phosphorylation (OXPHOS) (14). The Warburg effect is still to date of great interest to better understand tumor metabolism (15, 16). The description of the Warburg effect led over time to the deduction that mitochondria and OXPHOS play an inferior if any role in the energy consumption of tumor cells (17). It must however be noted that numerous malignancies with strongly proliferating cells are characterized by a high O_2_ consumption (OC), including HNSCC (18–20). Reports on an association of the OXPHOS pathway with a gene signature of recurrent oral squamous cell carcinoma (OSCC) support an important function of the OC in head and neck tumor and their progression (21). Consequently, genes associated with aerobic glycolysis and OXPHOS are both found up-regulated in malignant HNSCC cells with differences between classical and human papillomavirus (HPV)-associated tumors (19, 22).

High OC and insufficient re-oxygenation via a frequently leaky and poorly structured intratumoral neo-vasculature eventually result in oxygen depletion in tumor and surrounding tissues. This process enhances hypoxia along with the induction of transcription factors such as hypoxia inducible factors (HIFs) and the activation of signaling pathways. Hypoxia-related pathways impact numerous cellular functions such as apoptosis, necrosis, angiogenesis, the metastatic cascade via induction of epithelial-to-mesenchymal transition (EMT), and others (23–26). The knowledge of hypoxia markers has been used to assess surgical resection margins. Adjacent dysplastic tissue of HNSCC has shown an overexpression of hypoxia marker carbonic anhydrase 9 (CA IX), indicating high OC and insufficient O_2_ replenishment not only in the tumor tissue itself, but also in dysplastically altered surrounding tissue (27). High-grade dysplasia often occurs in peritumoral areas of HNSCC in the sense of field cancerization. Even though not yet invasively growing, these dysplasia need to be completely surgically removed due to the risk of the development of malignancy (28, 29). The fact that not only intratumoral, but also dysplastically altered peritumoral areas show signs of high OC and resulting hypoxia underlines the importance of adequate resection margins and the relevance of OC as a tool to optimize tumor margin recognition. Even though methods like immunohistology can provide relevant information on the tumor and its surroundings, *i.e.* the tumor microenvironment (TME), these methods are invasive to the tissue and require time-consuming preparations of the respective tissue. Non-invasively evaluating tissue oxygenation and OC via methods like fluorescent sensor foil technology could therefore bring further benefits to tumor diagnostics.

From the perspective of clinical management of patients with solid tumors, hypoxia resulting from high OC and poor re-oxygenation is recognized as a negative factor regarding therapeutical options. Particularly with respect to poor response to radiotherapy, hypoxia in tumor tissue has been the subject of previous and current research (30, 31). Hypoxia in tumor tissue is associated with poorer overall survival and poorer locoregional recurrence control, among others in HNSCC (32).

Similarly, the presence of a small safety margin during tumor resection, *i.e.* removal of the tumor with little surrounding healthy tissue, is associated with poorer overall survival. Such so-called “close margins” are usually defined as a resection margin of less than five millimeters (8). Ettl et al. described a 5-year overall survival in patients with head and neck tumors of 78% with a resection margin greater than five millimeters, but of only 51.7% when the resection margin was less than five millimeters (8). It is therefore of utmost importance to be able to clearly define the boundary between tumor and healthy tissue, preferably already intraoperatively.

The aim of the present study was to establish a high-resolution measurement of OC in HNSCC patients using a fluorescent sensor foil technology. This technology has been used to assess oxygen content in chronic wounds (33). Furthermore, it has been used to monitor the perfusion of free flaps after reconstructive surgery (34). The focus of this project was a time-dependent, comparative evaluation of oxygen consumption in macroscopically normal and malignant tissue. Therefore, following a feasibility assessment in a 3D co-culture model of carcinoma cells and fibroblasts isolated from the periphery of HNSCCs, proof-of-concept measurements of OC were conducted *in situ* in six HNSCC patients.

## 2 Materials and methods

### 2.1 Cell lines and cell culture

FaDu (hypopharyngeal carcinoma) and Kyse30 cells (esophageal squamous cell carcinoma) used in this study were purchased from DSMZ (Braunschweig, Germany). Peritumoral fibroblasts (PtFs) were isolated from HNSCC patient biopsies from mucosa macroscopically free of cancer (35).

To create red fluorescent FaDu and Kyse30 cells, parental cells were transfected with the pCAG-ef1-mCherry plasmid and stable cell lines were created by drug selection with 1 µg/ml puromycin.

Cells were grown in T25 or T27 cell culture treated flasks (Sarstedt AG, Nümbrecht, Germany) in an incubator at 37°C, 5% CO_2_ and 100% humidity, and passaged 1:5 to 1:20 (FaDu, Kyse30) or 1:4 (NF8) two to three times weekly. Kyse30 cells were grown in RPMI 1640 with L-Glutamine (Gibco, Dublin, Ireland) supplemented with 10% FCS and 1% Penicillin/Streptomycin (final concentration: 100 µg/ml, Gibco, Dublin, Ireland). For FaDu cells, DMEM (Gibco, Dublin, Ireland) supplemented with 10% FCS (FBS Superior, Sigma Aldrich, St.Louis, USA) and 1% Penicillin/Streptomycin (final concentration: 100 µg/ml, Gibco, Dublin, Ireland) was used. NF8 cells were grown in Fibroblast Growth Medium 2 (FGM-2) (C-23220, PromoCell, Heidelberg, Germany) supplemented with Supplement Mix 2 (C-39325, PromoCell).

### 2.2 3D co-culture model

Spheroids from red fluorescent FaDu and Kyse30 cells were generated by seeding 1000-20.000 cells per well into BIOFLOAT 96-well U-bottom plates (F202003, faCellitate, Mannheim, Germany) and leaving the spheroids to form over 72 hours. Next, spheroids were embedded into a type I collagen matrix. First a layer of 250 µl type I collagen (diluted to 1.5 mg/ml in imaging medium (RPMI w/o phenol-red, 10% FBS, 1% Penicillin/Streptomycin), and 0.5% 1M NaOH) was pipetted into the inner well of an ibidi 35mm glass bottom dish (81218-200, Ibidi GmbH, Gräfelfing, Germany) and allowed to form a gel at 37°C for 30 minutes. Two to five spheroids were carefully placed on top of the collagen layer and covered with 200 µl type I collagen at 1.5 mg/ml mixed with 2x10^5^ NF8 cells. After one hour at 37°C, 1.5 ml FGM was added, and the matrix-embedded co-culture was incubated over night at 37°C and 5% CO_2_.

For generating z-stacks of type I matrix-embedded spheroids, 10 µg/ml fluorescein (F6377, Sigma-Aldrich, St. Louis) was added to the 1.5 mg/ml collagen I solution. Z-stacks were acquired using the z-stack function of the LASX software and a Leica SP8 confocal laser scanning microscope using a 10x objective and the 488 nm laser for excitation of fluorescein and a 540 nm laser for excitation of mCherry.

### 2.3 *In vitro* OC measurements

**Figure 1:**
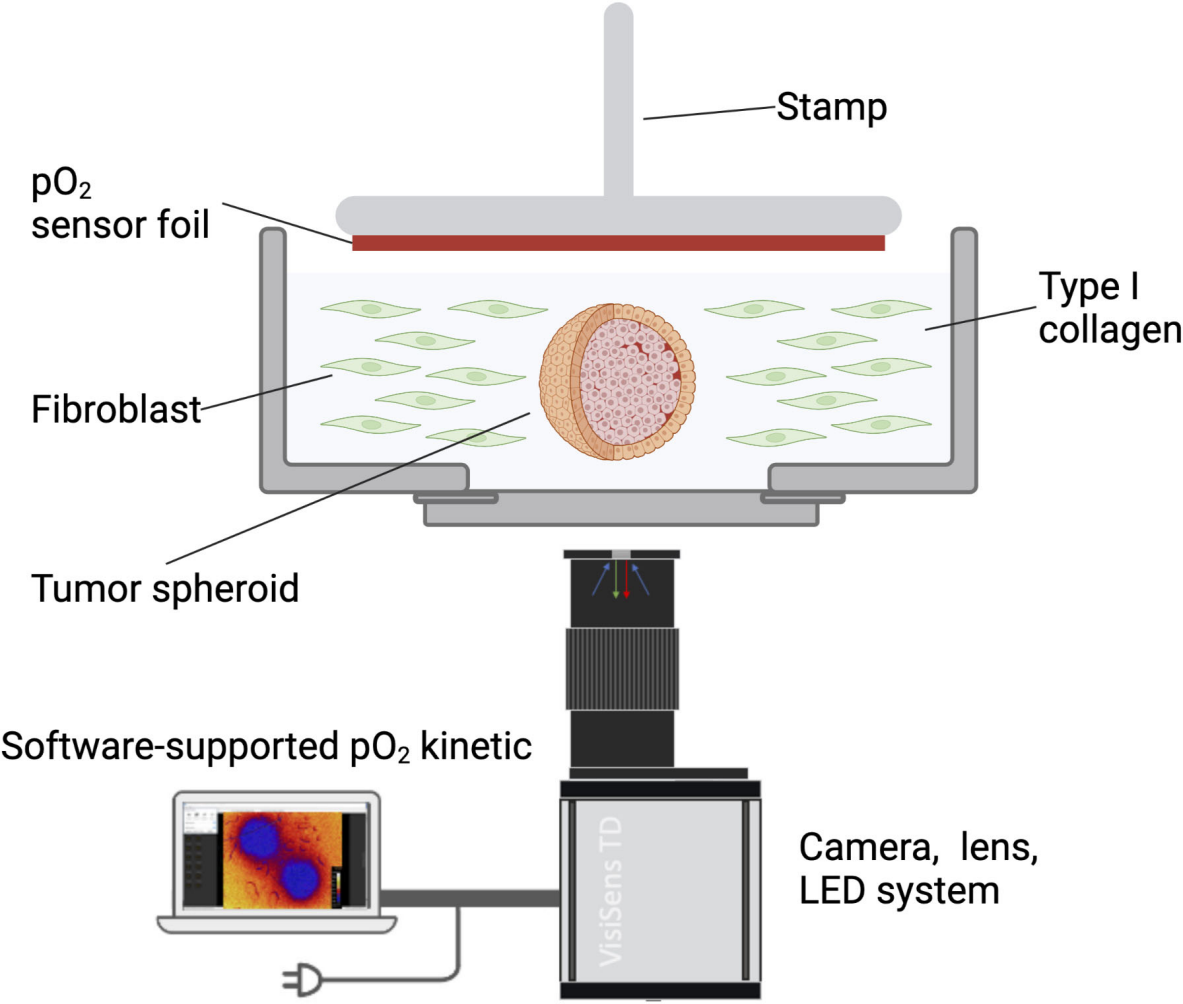
Schematic representation of the *in vitro* 3D model. Tumor cell spheroids were embedded in type I collagen containing HNSCC patient-derived fibroblasts. Oxygen consumption was measured with a sensor foil and an LED/camera system and recorded using the VisiSens software.

Before OC measurement, FGM was replaced with 1.5 ml of above-mentioned imaging medium. Measurements were performed using the STOp-Q method, as described previously (36), including the VisiSens TD Mic (PreSens Precision Sensing Technology GmbH, Regensburg, Germany) readout unit for microscopic O2 imaging. Ibidi dishes containing the spheroids embedded in type I collagen and NF8 cells were inserted into 6-well plates to fit into the imaging apparatus. Microscopic images over the entire area containing the spheroids and collagen/cell matrix were acquired using the tile scan function of the LASX software and a Leica DMi8 widefield fluorescence microscope (Leica, Wetzlar, Germany). Images were acquired with a 5x objective in the TXR channel (Excitation: 560/40, Emission: 639/75) and phase contrast. After a one-hour equilibration at 37°C in the incubator containing the STOp-Q device, measurements were performed in 10 second intervals for one hour using an exposure time of 150 ms (**Figure 1**).

### 2.4 Software, data analysis and statistics

Oxygen heatmaps for the *in vitro* measurements were exported from the VisiSens VS software after noise correction, which is conducted using a gaussian blur image filter with a kernel box size of 3*3 pixels on the processed delayered RGB image, and a 2*2 pixel software binning on the overall analyte image.

Microscopic images were processed by adjusting brightness/contrast, size and adding scale bars using Fiji (“FIJI Is Just ImageJ”) (37). Figures were generated using Inkscape (www.inkscape.org).

### 2.5 *In vivo* measurements

OC was measured *in vivo* in tumors of the head and neck area and macroscopically healthy tissue of patients that were treated at the department of otorhinolaryngology of the university hospital of LMU Munich. Six patients diagnosed with tumors of the head and neck area, four males and two females, were enrolled in this proof-of-concept study. Three patients suffered from lateral tongue carcinoma and three patients from carcinoma of the floor of the mouth. For detailed clinical and demographic parameters of all patients see **Table 1**. All examined tumors were HPV-negative tumors with a T-status ranging from T1 to T4. As a common risk factor, all patients smoked cigarettes and four of the six included patients drank alcohol daily. Two patients suffered from secondary carcinoma and had already undergone surgical tumor resection and adjuvant radiochemotherapy several years before. The macroscopically normal mucosa that was measured in conjunction with tumor areas for each patient were located several centimeters apart from the tumor areas. With measurements of lateral tongue carcinoma for example, normal mucosa was measured from the opposite side of the tongue. With measurements from the floor of the mouth, normal mucosa was measured either from a macroscopically healthy area from the floor of the mouth or – if the tumor consumed a large part of the floor of the mouth – from parts of the tongue.

**Table 1:** Patient profiles, clinical characteristics of patients included into the study (n=6).

Patient	Age	Sex	Tumor type	TNM	Therapy	Risk factors	HPV status	Additional information
1	78	f	lateral tongue carcinoma	pT1 pN0 L0 V0 Pn0 M0 R0 G2	transoral tumor resection, ipsilateral neck dissection	nicotine	negative	
2	58	m	lateral tongue carcinoma	pT2 pN0 L0 V0 Pn1 M0 R0 G3	transoral tumor resection, ipsilateral neck dissection	alcohol, nicotine	negative	
3	65	m	floor of mouth carcinoma	cT4 cN0 M0 G3 with infiltration of mandible	systemic therapy (pembrolizumab)	alcohol, nicotine	negative	oropharyngeal carcinoma 2011, pT3 pN0 M0 G2 R0, tumor resection, bilateral neck dissection, tracheostomy, radial flap, adjuvant radiochemotherapy
4	65	f	floor of mouth carcinoma	pT1 pN0 L0 V0 Pn0 M0 R0 G2	transoral tumor resection, bilateral neck dissection, tracheostomy	alcohol, nicotine	negative	
5	56	m	floor of mouth carcinoma	pT2 pN0 L0 V0 Pn0 M0 R0 G2	transoral tumor resection, bilateral neck dissection, tracheostomy	alcohol, nicotine	negative	
6	62	m	lateral tongue carcinoma	pT3 pN0 L0 V0 Pn0 M0 R0 G2	transoral tumor resection, bilateral neck dissection, tracheostomy, radial flap	nicotine	negative	oropharyngeal carcinoma 2013, pT1 pN2b L1 V0 M0 G3 R0, tumor resection, bilateral neck dissection, adjuvant radiochemotherapy

f, female; m, male.

Measurements were performed either in an intraoperative setting before tumor biopsy/resection or preoperatively. All measurements were performed after detailed patient information and verbal and written consent of the patients. The project was in accordance with the ethical standards of the institutional ethics committee of the Medical faculty of the LMU Munich (project 20-244) and with the 1964 Helsinki declaration and its later amendments or comparable ethical standards.

Luminescence imaging of oxygen was performed with the VisiSens 2D imaging System A1 (PreSens Precision Sensing Technology GmbH, Regensburg, Germany). The imaging setup including samples, sensor foil, and camera/lens is schematically depicted in results section. A handheld device to record the datasets and sensor foils for measuring oxygen consumption were used. The sensor foils were cut in adequate size and shape with sterile scissors (Fuhrmann GmbH, Bövingen 139, Munich, Germany), to fit on the respective regions of interest (tumor and healthy tissue). Data recording and evaluation was performed via the VisiSens AnalytiCal software (PreSens, Regensburg, Germany) provided with the systems.

## 3 Results

### 3.1 Discrimination of cancer cell spheroids and fibroblasts in a 3D *in vitro* co-culture model

In a comparison of esophageal and HNSCC cell lines comprising Kyse30, FaDu, Cal27, and Cal33, Kyse30 and FaDu showed the highest oxygen consumption rates (OCR) using the STOp-Q technology. Furthermore, high OCR for these two cell lines were confirmed using the Seahorse technology and were significantly higher than primary human nasal epithelial cells that served as normal controls (36). We opted for these two cell lines to provide optimal measurement conditions and thus allow for the highest sensitivity in the present 3D reconstruction model (**Figure 1**).

**Figure 2:**
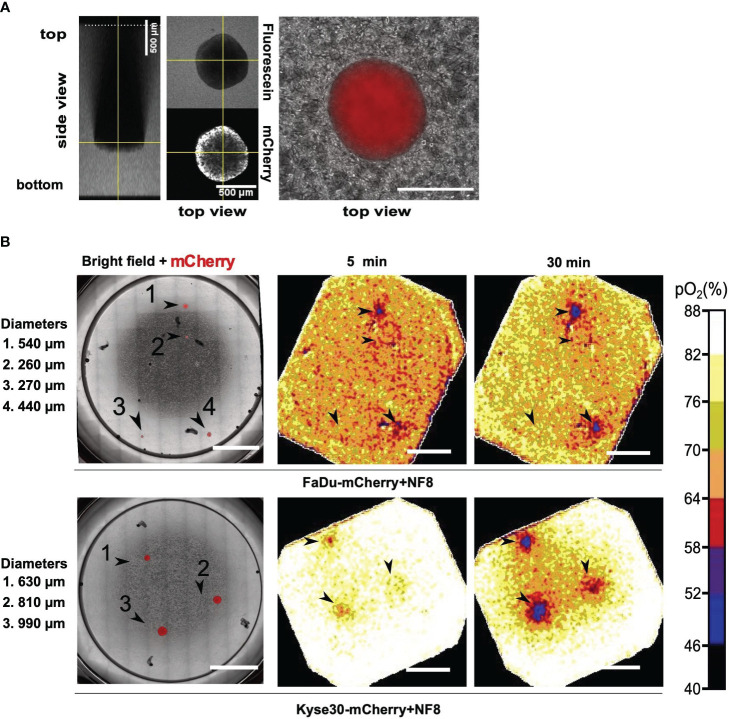
Oxygen consumption measurements in a 3D *in vitro* tumor model. **(A)** Z-stack of a FaDu mCherry expressing spheroid in a fluorescein labelled collagen I matrix. Left image represents side view, central and right images represent top view. Overlay of mCherry signal and phase contrast of a Kyse30 mCherry expressing spheroid within a collagen I and fibroblast (NF8) matrix are shown in the right panel. **(B)** Overlay of mCherry signal and phase contrast of FaDu/Kyse30 mCherry expressing spheroids within a collagen I and NF8 matrix. Spheroid diameters are indicated on the left (left panels). Oxygen heatmaps of the area shown in the microscopic images after 5 and 30 minutes of oxygen measurement (middle and right panels). Data of FaDu cells are depicted in the top row, data for Kyse30 cells in the bottom row. Arrowheads indicate the location of spheroids. Scale bars: 5 mm. Color bar indicating oxygen concentrations are shown on the right. Shown are representative images of n = 3 independent experiments performed with multiple spheroids for each cell line.

PtFs were used in the present model to incorporate peritumoral fibroblasts as a major cell type of non-malignant stromal cells and thereby mimic closely the situation *in vivo*. Tumor spheroids were visualized based on the constitutive expression of mCherry fluorescent protein following stable transfection (**Figures 2A, B**). To measure the thickness of the collagen matrix and the localization of tumor cell spheroids, the matrix was stained using fluorescein and z-stacks were acquired by confocal laser scanning microscopy. The total thickness of the matrix was approximately 2.5 mm, and the distance from the spheroid to the surface of the matrix was approximately 1.5 mm (**Figure 2A**, left panels).

Oxygen measurements were performed over a time period of one hour in ten second intervals and with an exposure time of 150 ms using the STOp-Q technology (36). Oxygen heatmaps depicted in **Figure 2B** represent the oxygen concentration after 5 minutes and 30 minutes of measurement, respectively. FaDu cell spheroids embedded in type I collagen and PtF matrix were detected down to a diameter of 440 µm within 5 minutes of measurement. Smaller spheroids were not detected even after 30 minutes of measurement (**Figure 2B**; **Figure S1**). Under identical conditions, Kyse30 cell spheroids with a minimum diameter of 630 µm were detected after five minutes of measurement (**Figure 2B**; **Figure S2**). After 30 minutes also spheroids with a diameter of 440 µm were identified based on OC measurements (**Figure S2**).

In summary, STOp-Q-based OC measurements allowed the spatial discrimination of tumor cell spheroids from surrounding stromal fibroblasts in ECM. Hence, 3D tumor cell depositions in the sub-millimeter range were detected based on their oxygen consumption and could be distinguished from fibroblasts in a type I collagen-based matrix.

### 3.2 *In situ* OC measurement of tumor tissue and healthy mucosa in HNSCC patients

**Figure 3:**
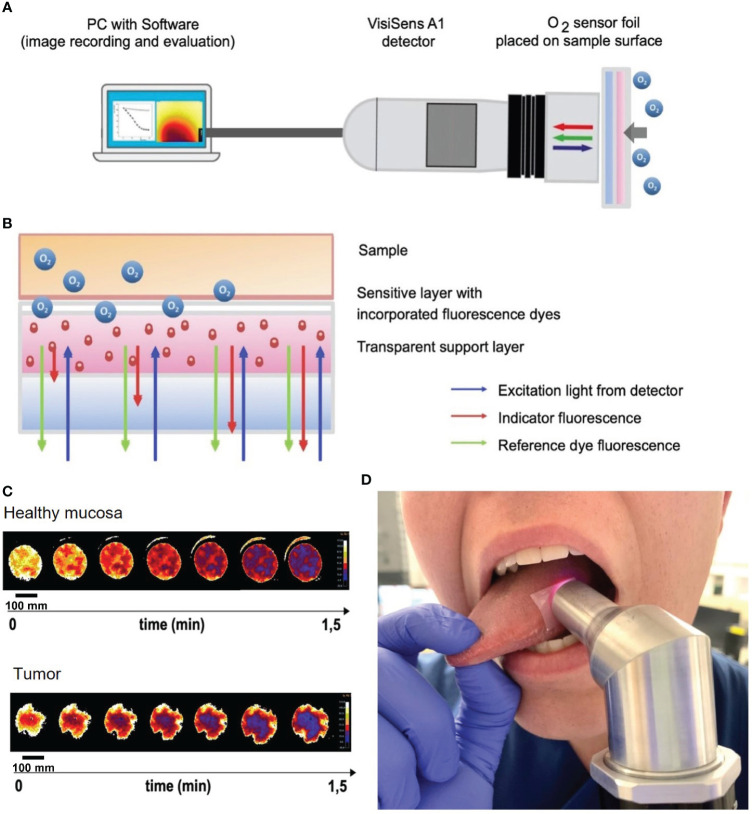
Oxygen consumption rate measurements *in situ* on HNSCC of the tongue and mouth. **(A)** Schematic of the VisiSens A1 detector camera connected to a laptop with the VisiSense software for image recording and evaluation. The handheld camera records the oxygen consumption rate measured by the sensor foil that is placed on the tissue (tumor and healthy mucosa). **(B)** Schematic of the measurement principle of the used sensor foils. The foil has two components: a sensitive layer with incorporated fluorescence dyes and a transparent support layer. The foil is placed on the tissue with the sensitive layer facing the tissue. Tissue oxygen and with it the OC are detected by a shift in the indicator fluorescence signal of the sensor foil. The reference dye fluorescence signal emits a steady signal. The excitation light derives from the detector camera. **(C)** Heatmaps of oxygen consumption of a representative patient showing measurements of healthy mucosa (upper image) and tumor (lower image). Note, that peripheral areas with continuously high O_2_ values are a result of fresh oxygen at the border of the handheld camera entering via the air and have been excluded from the measurements. Scale bars: 100 mm. **(D)** Example of *in situ* measurement of mucosa of the lateral tongue with the sensor foil and detecting camera.

Measurement of OC was performed as described in detail in the Materials and Methods section for n = 6 HNSCC patients. The sensor foil placed on the sample surface contains a red fluorescent oxygen indicator dye, a stable green reference fluorescent dye, embedded in an oxygen-permeable and transparent membrane. The red indicator fluorescence decreases with increasing oxygen content, whereas the green fluorescence remains stable regardless of changes in oxygen content and serves as reference. The red-to-green fluorescence signal ratio informs about the oxygen content of tissue and in time dependence also about the OC. The detector unit is a small handheld USB-powered camera system with incorporated excitation LEDs and fluorescence filters. The excitation LEDs illuminate the respective sensor foil with blue light and excite the luminophores that are enclosed in the sensitive layer of the foil. Optical filters separate excitation and emission light. The sensor foils emit red and green signals (indicator signal and reference signal, respectively), which are collected in the wavelength-separated red and green channels of the RGB camera detector and stored in a RGB color image (**Figures 3A, B**).

Sensor foils were carefully applied to the regions of interest of all six HNSCC patients without pressure to avoid tissue hypoxia due to external application of pressure onto the tissue, but firmly enough to avoid fresh air – and with that, ambient oxygen – to reach the sensor foils while measuring. With such a setup, the camera creates an airtight seal, so that only the tissue oxygen consumption is measured without any replenishment from the surrounding oxygen. Due to the uneven surface of tumor tissue with possible exulcerations, the adhesion of the sensor foils and the contact of the handheld device with the sensor foils and the tissue underneath can be more difficult than with evenly flat mucosa. This might alter the measurements due to difficulties in creating an airtight seal. Therefore, areas where proper adhesion of the sensor foils was impossible were excluded from the measurements. *In situ* measurements were performed in three second intervals with a maximum measurement time of two minutes. Data recording and evaluation was performed via the VisiSens analytical software (PreSens, Regensburg, Germany) provided with the system.

The detector was placed on the O_2_ sensor foil, which was applied on the area of interest (*i.e.*, normal mucosa or carcinoma areas). OC were measured in tumor and macroscopically normal mucosa of the tongue or floor of the mouth in all six patients. **Figure 3D** shows a representative picture of an *in situ* measurement of mucosa of the lateral tongue with the sensor foil and detecting camera. Oxygen heatmaps depicted in **Figure 3C** represent the oxygen consumption of healthy mucosa (upper panel) and tumor (lower panel) in one exemplary measurement of one of the six measured patients in this study. Heatmaps start with bright yellow colors, indicating a high oxygen content in the tissue. The darker the color of the heatmaps develop, the lower the oxygen content in the tissue gets over time, with deep blue colors at the end of the measurement indicating the lowest oxygen content of the measurement over time.

**Figure 4:**
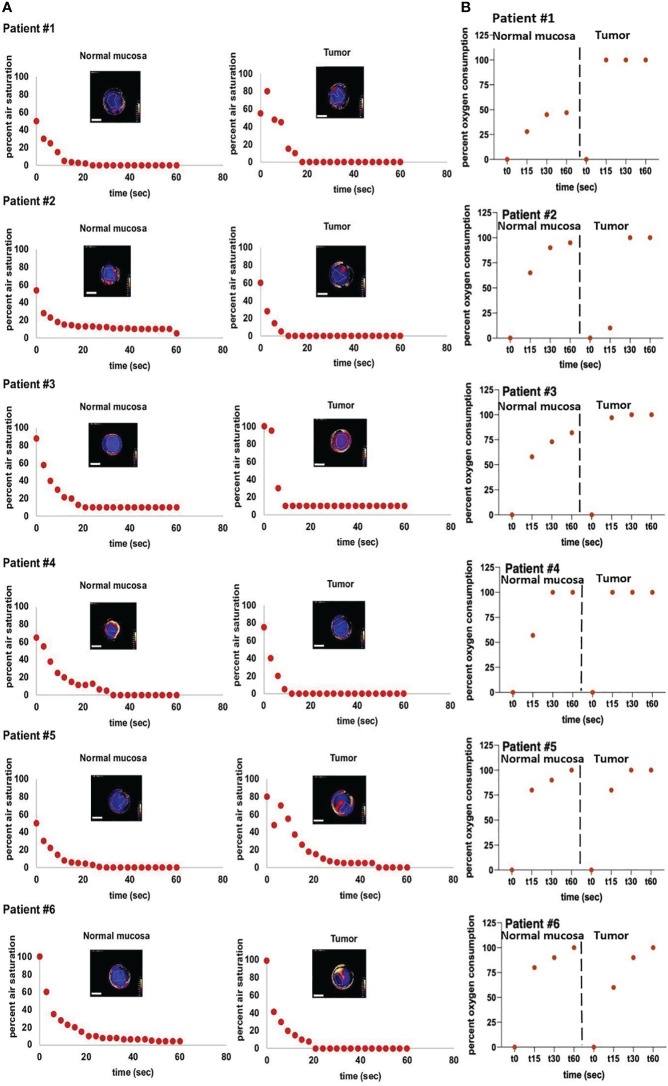
*In vivo* measurements of air saturation and OC-rates. Measurements of air saturation and oxygen consumption of normal mucosa und tumor tissue of six individual patients. **(A)** depicts the raw measurement points, meaning a drop in oxygen (y-axis) over a time span of 60 seconds (x-axis) of normal mucosa (left panels) and tumor (right panels). All depicted heatmaps represent the last measurement of each individual measurement. Areas with poor adhesion of the sensor foil to the tissue were excluded from the measurements. Note, that peripheral areas with continuously high O_2_ values are a result of fresh oxygen at the border of the handheld camera entering via the air and have been excluded from the measurements. Scale bars: 10 mm. **(B)** depicts the oc-rate (y-axis) of the same patients (normal mucosa: left panels, tumor tissue: right panels) over a time span of 60 seconds (x-axis). The OC-rate starts at 0, indicating no consumption of oxygen at the beginning of the measurement and ends at a maximum value of 100%, indicating that all available tissue oxygen of the respective region of interest (ROI) has been consumed. For clearer separation, measurements of normal mucosa and tumor tissue are separated by dotted lines. In summary, **(B)** depicts the conversion from the raw data of **(A)** into oxygen consumption over time.

**Figure 5:**
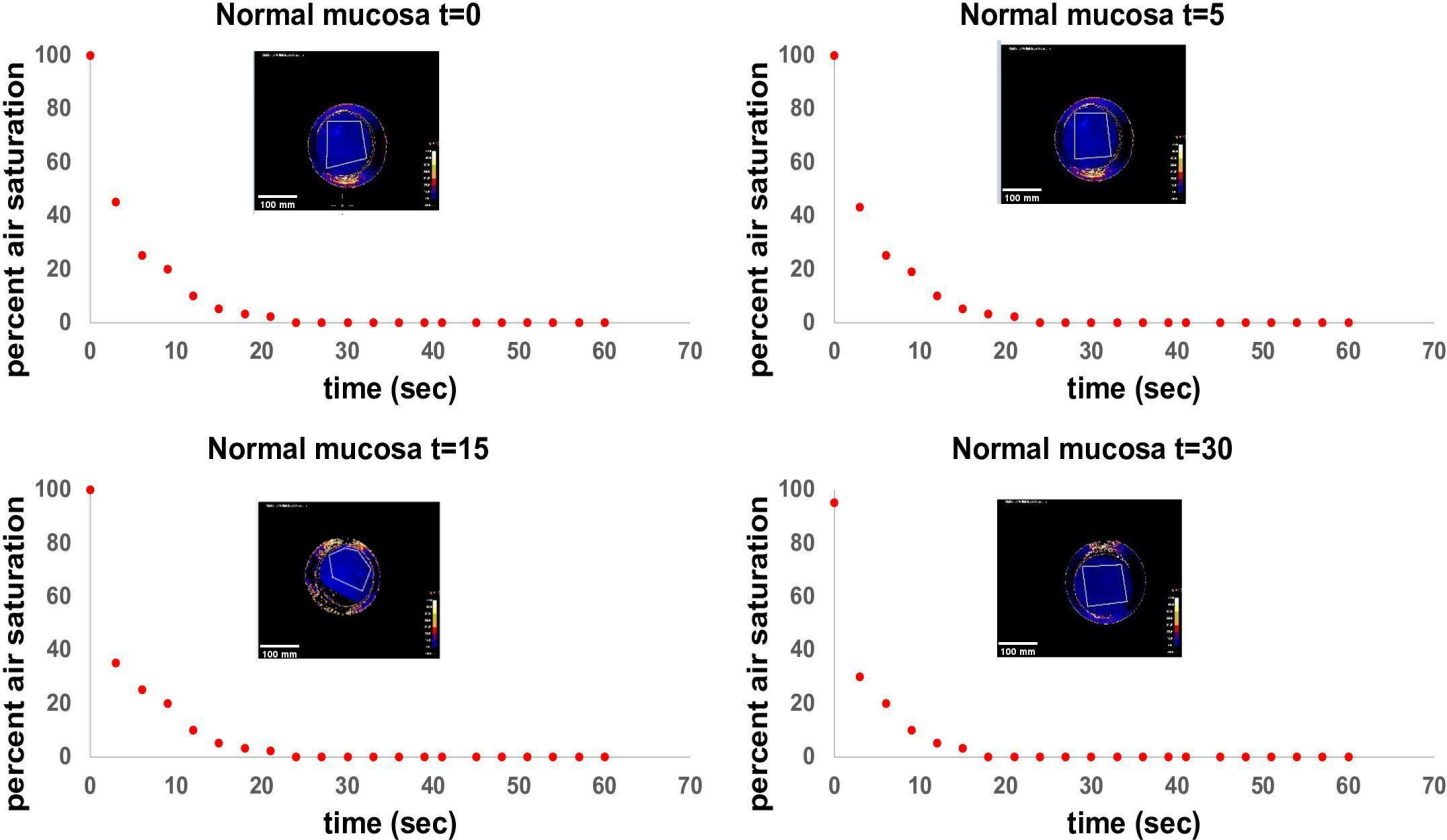
Standardized measurement of OC in healthy mucosa at different time points. T=0 as first measurement, t=5 after 5 minutes, t=15 after 15 minutes and t=30 after 30 minutes in total. Measurements were performed in a healthy volunteer in the same area of the tongue (anterior part of the lateral tongue). The volunteer did not eat or drink in between the measurements, nor did they talk excessively or were otherwise physically active. Time in seconds is shown on the x-axis, air saturation in per cent is shown on the y-axis. Every point on the diagram represents a measurement point at an interval of three seconds each. The heatmap in each diagram represents the ending point of the measurement after 60 seconds. Note, that peripheral areas with continuously high O_2_ values are a result of fresh oxygen at the border of the handheld camera entering via the air and have been excluded from the measurements. Scale bars: 10 mm.

Quantified OC of tumor and healthy tissues were plotted against time and standardized to the initial O_2_ percentage (**Figure 4**). Both, healthy mucosa, and tumor areas showed a measurable consumption of oxygen within the time frame of the assessment. Oxygen partial pressure levels in normal mucosa gradually decreased over time and reached values below 5% air saturation (7.46 torr) after approx. 48 seconds on average. Oxygen levels in tumor areas dropped sharply to levels below 5% air saturation within 15 seconds of measurement on average and stayed at this low level throughout the remaining measurement time. Differences of OC between healthy mucosa and tumor areas were analyzed for statistical significance with a two-sample t-test assuming different variances. With a p-value of 0,01168221 at a time point of 15 seconds into the measurement and a p-value of 0,01623259 at a time point of 30 seconds into the measurement, the differences between OC in tumor tissue versus healthy tissue were significant. 60 seconds into the measurement, most of the readings leveled off, so that a stable oxygen partial pressure value was reached. Interestingly, at this time point, all oxygen consumption values had reached almost 100% for carcinomas, whereas values for normal mucosa as low as < 50% were observed in patient #1 (**Figure 4**). Of note, measurements of the two patients that had suffered from secondary carcinoma and had undergone adjuvant radiochemotherapy several years back showed the lowest OC at t15 in tumor tissue (patients 2 and 6), but not in healthy tissue. In accordance with all other measurements of tumor though, these two measurements eventually leveled off at 100% OC after 60 seconds, too. Hence, these relevant differences can only be seen early into the measurements. Not all measurements started at 100% O_2_ saturation. However, the intra-individual measurements of each patient showed repeatedly steady starting points of O_2_ saturation, be it at 100% or below. In addition, the measurements of tumor and healthy tissue in each patient had similar saturation starting points. We believe that the differences in starting O_2_ saturation has to do with external factors like the patients’ body temperature and others. To demonstrate the temporal reproducibility of the measurements, we performed measurements of healthy mucosa in one volunteer at different time points (t=0 as first measurement, t=5 after 5 minutes, t=15 after 15 minutes and t=30 after 30 minutes in total, see **Figure 5**). The volunteer did not eat or drink in between the measurements, nor did they talk excessively or were otherwise physically active.

In conclusion, significant differences of OC were detected in matched pairs of normal mucosae and HNSCC *in situ*.

## 4 Discussion

Physiological homeostasis of oxygen supply and consumption assures balanced metabolic rates. In tumor tissue, however, this homeostasis frequently gets out of balance and oxygen consumption exceeds the available supply. A high OCR, thus, can result in a hypoxic TME and tissue hypoxia (20). In solid tumors, hypoxia has been recognized as a negative prognostic factor regarding therapeutical options, particularly regarding radiotherapy (30, 31). Subpopulations of tumor cells can survive in hypoxic conditions and play a major, unfavorable role in resistance to irradiation (30). Hypoxic conditions in the TME occur in several solid tumors (38–40). For HNSCC, tumor hypoxia is associated with a poorer overall survival and poorer locoregional recurrence control (41). Upregulation of the transcription factor (TF) GATA binding protein 3 (GATA3) in HNSCC leads to an inhibition of degradation of the TF HIF-1α and thereby creates hypoxic conditions in the TME (41). As a counter-regulating mechanism observed in HNSCC, hypoxic conditions induce the upregulation of a nucleotide excision repair protein, DDB2 (damaged DNA binding protein), which represses the transcription and expression of HIF-1α and other markers of hypoxia (42).

Hence, not only can hypoxia be used as a potential target in cancer therapy, but the visualization of OCR as a sign of hypoxia is of great diagnostical and therapeutic interest. This notion is further supported by reports on the implication of OXPHOS in tumor recurrences in HNSCC (21) and as metabolic vulnerability in highly aggressive triple-negative breast cancer that fail to respond to chemotherapy (43). OXPHOS also represents an attractive target for therapeutic strategies targeting cancer stem cells, which may preferentially rely on this energy source, for example in ovarian cancers, therapy-resistant metastatic breast cancer, and others (44). However, to reliably distinguish malignant from healthy tissue using pO_2_, it is mandatory to perform measurements *in situ* with high spatiotemporal resolution. In contrast to systems based on electrodes or soluble sensors, immobilized O_2_-sensitive fluorophores - as used in this research project – are deemed more suitable for such application (45). Main advantages of a setup with sensor foils in combination with a handheld camera are (a) that no preparation or treatment of the tissue is required, (b) measurements can be performed *in situ* in a time frame of a few minutes, and (c) topological assessment with high resolution is obtained. Sensor foils with immobilized fluorophores can be safely used on skin and mucosa without the harm of possible toxicity of applying soluble sensors. Another advantage is the spatiotemporal resolution in the sub-millimeter range as shown *in vitro* in the 3D co-culture tumor model presented in this study. These measurements showed promising results with a detection of tumor cell spheroids with two cell lines that had shown high OCR in previous studies via the STOp-Q method down to a diameter of 440 µm. It must be noted that FaDu (hypopharynx) and Kyse30 (esophagus) differ in their original sub-localization from the primary tumors measured *in vivo*. Although differences in the actual size limit of detection between *in vitro* and *in vivo* settings might result partly from malignant cells of varying sub-localization, the choice of upper aerodigestive tract carcinoma cell lines with higher OCR enabled us to provide detection limits under optimized conditions. It must however be noted that altered detection limits may result with additional cell lines and, even more so, *in situ*. In the latter situation, measurements may by further hindered by saliva, topology, and accessibility. The measurement of oral cavity cancers was designed as a proof-of-concept study with easily accessible malignancies under non-surgical conditions. Future aims comprise the transfer of this technology to intraoperative measurements of residual tumor cells to provide optimized tools for R0 resections.

For the case of disseminated tumor cells in lymph nodes, isolated tumor cells (ITCs), micrometastases, and (macro)metastases are distinguished according to size. ITCs are regarded as metastatic lesions of less than 0.2 mm and are staged pN0. Micrometastases are 0.2-2.0 mm in size and are staged pN1mi, whereas macrometastases, also termed metastases, are larger than 2.0 mm. Assimilating these criteria for residual tumor cells in the vicinity of primary cancers, OC measurements using sensor foils would potentially allow the detection of micro-/and macrometastases and could therefore help eradicate these malignant clusters during surgical procedures (46).

*In situ* measurements on a small cohort of HNSCC patients corroborated our *in vitro* results and allowed a distinction of matched normal epithelia from cancer tissue via OC measurements over a time frame of few seconds (*i.e.*, 15 s). To our knowledge, this is the first implementation of measurements of OC over a defined timeline of HNSCC *in situ*. Since pO_2_ is a metabolic parameter of high clinical and prognostic value, devices that allow standardized and easy measurement of this parameter could represent a significant benefit for patients. Based on the proof-of-concept described in the present study, tissue imaging via sensor foils with immobilized fluorophores qualifies as a potential diagnostic imaging tool due to its high spatiotemporal resolution of tumor aggregates at the micrometastasis size range. Such measurements can be easily integrated in an intraoperative workflow as a non-invasive, rapid procedure to facilitate decisions concerning surgical margins intraoperatively. This could result in a higher treatment safety for the individual patient in terms of sufficient resection margins. Further projecting into the future, OCR measurements might be used as planning tools to detect optimal surgical margins prior to surgery. If a spatial resolution in the micrometer range could be reliably reached *in vivo*, the surgeon might have support in resecting the respective tumor oncologically as safe as possible and functionally as beneficial as possible.

Limitations to this study are the small cohort size and heterogeneity of the tumor stages within the cohort. Larger cohorts of patient samples with different tumor stages (T1-T4 after TNM classification) could give more information on tumor stage-specific OC and could strengthen the theory of higher OC in tumor tissue with higher statistical impact. Another limitation to the application is the handling and adherence of the sensor foils. The foils are placed onto the tissue manually; after placing the sensor foil onto the tissue, the handheld camera is placed onto the sensor foil. An incorporation of the foil into the handheld camera would simplify the measurement process. Additionally, measuring oropharyngeal tissue is always accompanied by the possibility of saliva, mucus or blood being trapped between tissue and sensor foil and influencing the measurements. The areas of interest were tapped dry before applying the sensor foils, but this remains a possible source of error. Furthermore, it must be mentioned that the measurements of different patients showed different starting values of O_2_. This must be critically evaluated. Different tissue temperature or moisture may alter the onset of the measurement. The standardized measurement of healthy mucosa at different time intervals in a volunteer as depicted in **Figure 5** also shows a minimal change in the starting value of O_2_ in the last measurement at 30 minutes (90% at t=30 vs 100% at earlier time points). This might indicate a possible influence of a slight alteration in temperature of the tissue over time on pO_2_ measurement. In further experiments, possible factors that could alter the measurements need to be thoroughly evaluated and considered. Since this was a proof-of-concept study, further experiments will also focus on improvements in the execution of the experiments *in situ*.

Two of the six patients included in this study had undergone radiochemotherapy several years back for the treatment of oropharyngeal carcinoma. These two patients showed the lowest OC in tumor tissue of all patients at early time points of measurement. This is in line with previous studies that described differences in oxygenation of irradiated compared to non-irradiated skin. Auerswald et al. showed significantly decreased oxygen levels of chronic wounds of irradiated human skin *in vivo* compared to intact, non-irradiated skin (33). Therefore, a time-dependent measurement of oxygen consumption may provide information on the behavior of not only tumor tissue but generally on tissue that has undergone extensive treatment like radiotherapy.

## 5 Conclusion

In this project, we present an approach to distinguish tumor tissue of the head and neck area from healthy tissue via measurements of the oxygen consumption (OC). In an *in vitro* model, we performed a detection of tumor cell spheroids in a type I collagen matrix containing stromal cells down to a diameter of 440 µm with the STOp-Q-method, showing the high spatiotemporal resolution of this method. An *in vivo* proof-of-concept study concentrated on the distinction between matched healthy and tumor tissue of the head and neck area in oral cancers that were easily accessible in a pre- and intraoperative setting. We found a significantly higher OC in tumor tissue compared to macroscopically normal tissue. This approach harbors great clinical potential regarding an *in vivo* differentiation of normal and malignant tissue, especially with respect to resection margins.

## Data availability statement

The original contributions presented in the study are included in the article/**Supplementary Material**. Further inquiries can be directed to the corresponding author.

## Ethics statement

The studies involving humans were approved by the medical faculty of the LMU Munich (Ludwig-Maximilians-University Munich) (project 20-244). The studies were conducted in accordance with the local legislation and institutional requirements. The participants provided their written informed consent to participate in this study.

## Author contributions

MS performed *in situ* measurement, generated figures, and wrote the manuscript; AS performed *in vitro* measurement, generated figures, and wrote the manuscript; GL and RM provided foils and measurement devices, and support in data analysis; MW, MC, and FH enrolled patients; MC helped writing the manuscript; OG and FH coordinated the study, wrote the manuscript, and acquired funding. All authors contributed to the article and approved the submitted version.

## Supplementary material

Supplementary Figure 1 Oxygen measurements in a 3D *in vitro* tumor model with FaDu spheroids. Overlay of mCherry signal and phase contrast of FaDu mCherry expressing spheroids within a collagen I and NF8 matrix. Spheroid diameters are indicated on the left (left panels). Oxygen heatmaps of the area shown in the microscopic images after 5, 20 and 30 minutes of oxygen measurement (middle panels). Arrowheads indicate the location of spheroids. Scale bars: 5 mm. Color bar indicating oxygen concentrations on the right.

Supplementary Figure 2 Oxygen measurements in a 3D *in vitro* tumor model with Kyse30 spheroids. Overlay of mCherry signal and phase contrast of Kyse30 mCherry expressing spheroids within a collagen I and NF8 matrix. Spheroid diameters are indicated on the left (left panels). Oxygen heatmaps of the area shown in the microscopic images after 5, 20 and 30 minutes of oxygen measurement (middle panels). Arrowheads indicate the location of spheroids. Scale bars: 5 mm. Color bar indicating oxygen concentrations on the right.
