# Supplementary figures and images for: Quantification of oxygen consumption in head and neck cancer using fluorescent sensor foil technology
Source: Front Oncol. 2024 Feb 8;14:1002798. doi: 10.3389/fonc.2024.1002798 (PMC10882065; doi:10.3389/fonc.2024.1002798)

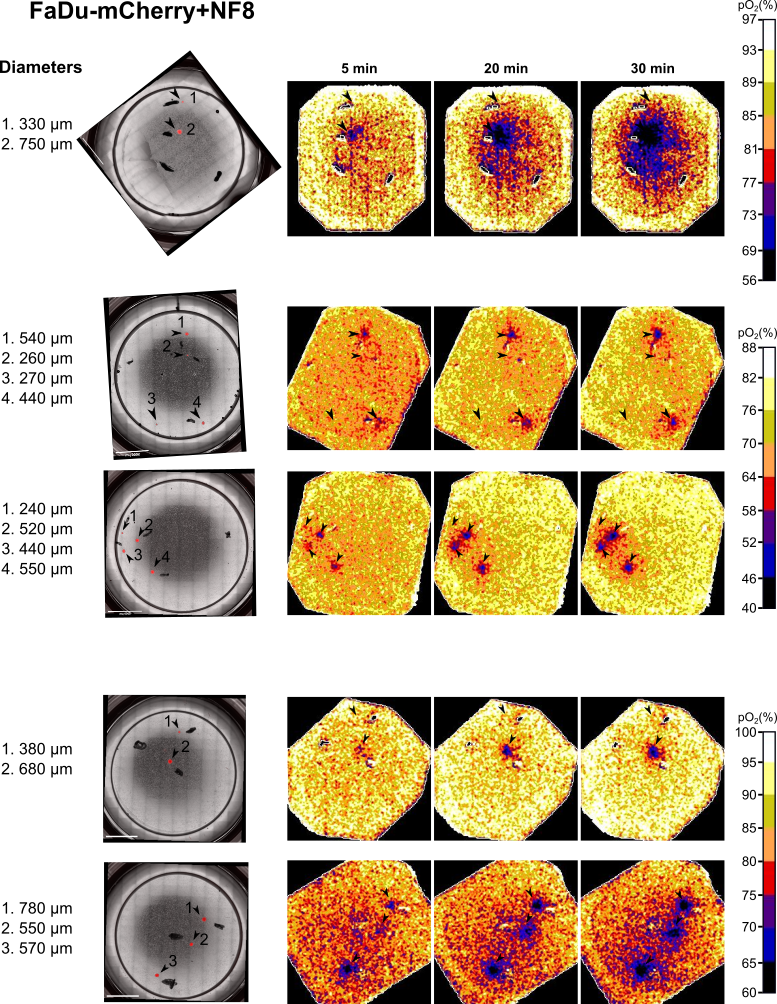

Supplement: Supplementary Figure 1 — Oxygen measurements in a 3D in vitro tumor model with FaDu spheroids. Overlay of mCherry signal and phase contrast of FaDu mCherry expressing spheroids within a collagen I and NF8 matrix. Spheroid diameters are indicated on the left (left panels). Oxygen heatmaps of the area shown in the microscopic images after 5, 20 and 30 minutes of oxygen measurement (middle panels). Arrowheads indicate the location of spheroids. Scale bars: 5 mm. Color bar indicating oxygen concentrations on the right. [file Image_1.png]

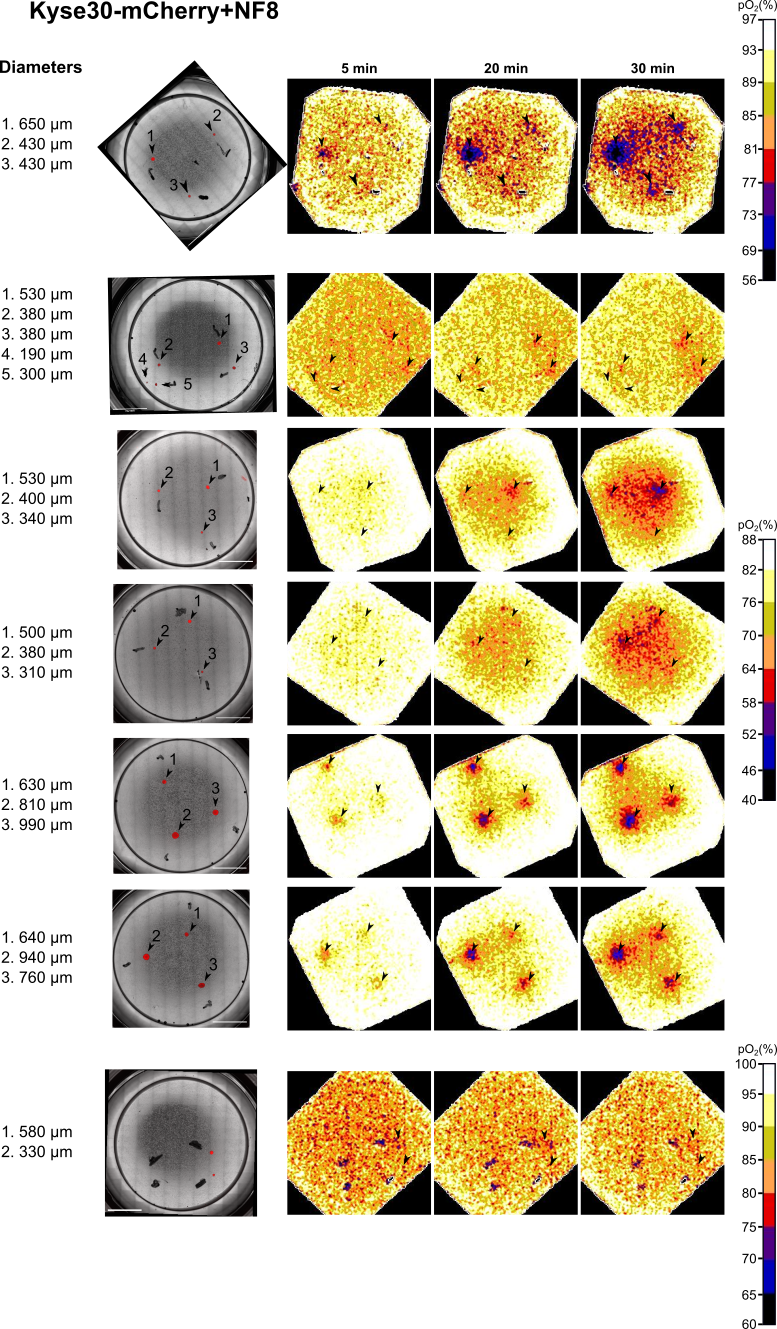

Supplement: Supplementary Figure 2 — Oxygen measurements in a 3D in vitro tumor model with Kyse30 spheroids. Overlay of mCherry signal and phase contrast of Kyse30 mCherry expressing spheroids within a collagen I and NF8 matrix. Spheroid diameters are indicated on the left (left panels). Oxygen heatmaps of the area shown in the microscopic images after 5, 20 and 30 minutes of oxygen measurement (middle panels). Arrowheads indicate the location of spheroids. Scale bars: 5 mm. Color bar indicating oxygen concentrations on the right. [file Image_2.png]
